# *OsTH1* is a key player in thiamin biosynthesis in rice

**DOI:** 10.1038/s41598-024-62326-2

**Published:** 2024-06-12

**Authors:** Maria Faustino, Tiago Lourenço, Simon Strobbe, Da Cao, André Fonseca, Isabel Rocha, Dominique Van Der Straeten, M. Margarida Oliveira

**Affiliations:** 1 Instituto de Tecnologia Química e Biológica António Xavier, Universidade Nova de Lisboa, 2780-157 Oeiras, Portugal; 2 Laboratory of Functional Plant Biology, Department of Biology, Ghent University, K. L. Ledeganckstraat 35, B-9000 Gent, Belgium; 3 Present Address: University of Geneva, Quai E. Ansermet 30, 1211 Geneva, Switzerland

**Keywords:** Molecular engineering in plants, Metabolic engineering, Functional genomics

## Abstract

Thiamin is a vital nutrient that acts as a cofactor for several enzymes primarily localized in the mitochondria. These thiamin-dependent enzymes are involved in energy metabolism, nucleic acid biosynthesis, and antioxidant machinery. The enzyme HMP-P kinase/thiamin monophosphate synthase (TH1) holds a key position in thiamin biosynthesis, being responsible for the phosphorylation of HMP-P into HMP-PP and for the condensation of HMP-PP and HET-P to form TMP. Through mathematical kinetic model, we have identified TH1 as a critical player for thiamin biofortification in rice. We further focused on the functional characterization of *OsTH1*. Sequence and gene expression analysis, along with phylogenetic studies, provided insights into *OsTH1* bifunctional features and evolution. The indispensable role of OsTH1 in thiamin biosynthesis was validated by heterologous expression of *OsTH1* and successful complementation of yeast knock-out mutants impaired in thiamin production. We also proved that the sole *OsTH1* overexpression in rice callus significantly improves B1 concentration, resulting in 50% increase in thiamin accumulation. Our study underscores the critical role of *OsTH1* in thiamin biosynthesis, shedding light on its bifunctional nature and evolutionary significance. The significant enhancement of thiamin accumulation in rice callus upon *OsTH1* overexpression constitutes evidence of its potential application in biofortification strategies.

## Introduction

**Figure 1:**
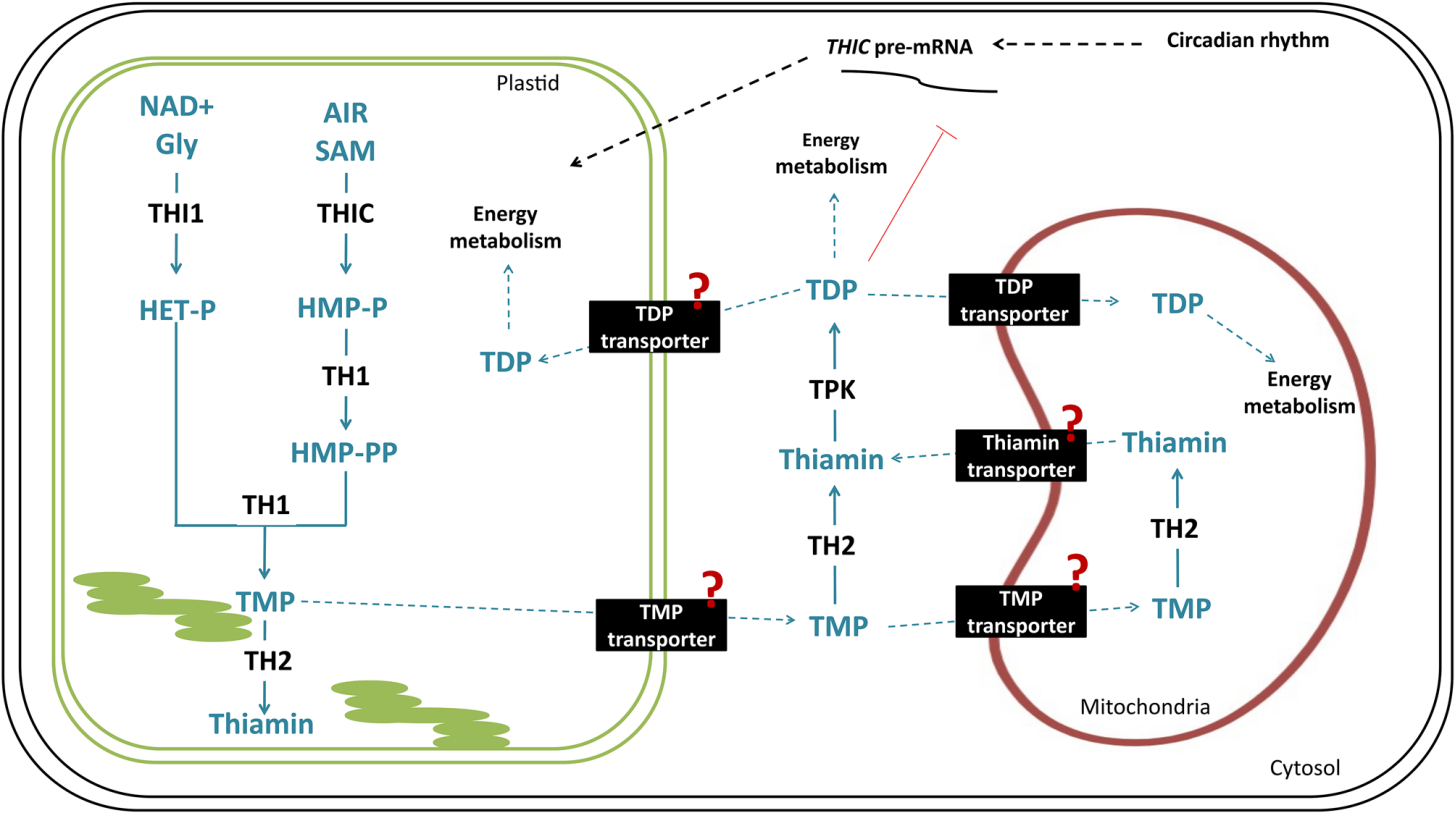
Thiamin biosynthesis in plants. Figure adapted from Strobbe and Van Der Straeten^17^. Biosynthetic pathway is depicted in blue, while enzymes are shown in black. Black boxes depict transporters, interrogation points represent unknown enzymes. Only one TDP transporter is known^22^; other transporters are proposed. The synthesis of the pyrimidine moiety (HMP-P) requires 5-aminoimidazole ribonucleotide (AIR) and S-adenosylmethionine (SAM) as substrates and is catalyzed by 4-amino-2-methyl-5-hydroxymethylpyrimidine phosphate (HMP-P) synthase (THIC)^6,7^. THIC mRNA precursor is regulated by a riboswitch in its 3’ untranslated region (UTR). This riboswitch functions as a TDP sensor controlling THIC mRNA stability and consequently, TDP production^23–26^. The synthesis of the thiazole moiety (HET-P) is mediated by 4-methyl-5-b-hydroxyethylthiazole phosphate (HET-P) synthase (THI1)^8,9^, the substrates of this reaction are nicotinamide adenine dinucleotide (NAD) and glycine^27^. TH1 (HMP-P kinase/thiamin monophosphate (TMP) pyrophosphorylase) phosphorylates HMP-P to HMP-PP and also condenses HMP-PP and HET-P to form TMP^3,10^. TH2 dephosphorylates TMP^5,11,12^ which is then pyrophosphorylated to TDP by TDP kinases (TDPK**s**)^13^.

Thiamin is a water-soluble vitamin whose main active form (thiamin diphosphate, TDP) acts, in all domains of life, as a cofactor for enzymes of the central carbon metabolism related to tricarboxylic acids and other acetyl Co-A pathways (e.g. fatty acids synthesis/breakdown, mevalonate dependent pathways), as well as amino acid pathways^1,2^. Since animals and humans cannot synthesize TDP, they must obtain this cofactor as free thiamin from plant sources^3^. Although plants are a primary source of dietary thiamin, little is understood about how plants synthesize this compound. The major forms of vitamin B1 present in the cells are free thiamin, thiamin monophosphate (TMP) and thiamin diphosphate (TDP)^4^. In plants, de novo biosynthesis of thiamin occurs predominantly in photosynthetic tissues and takes place in plastids, cytosol, and mitochondria^5^. The first steps of the biosynthetic pathway occur in the plastid by the formation of a pyrimidine moiety (HMP-P) and a thiazole moiety (HET-P) mediated by 4-amino-2-methyl-5-hydroxymethylpyrimidine phosphate (HMP-P) synthase (THIC)^6,7^ and 4-methyl-5-b-hydroxyethylthiazole phosphate (HET-P) synthase (THI1)^8,9^, respectively (Fig. 1). In the plastid, HMP-P is phosphorylated into HMP-PP by the action of the bifunctional HMP-P kinase/TMP synthase (TH1) which subsequently condenses HMP-PP and HET-P to form thiamin monophosphate (TMP)^3,10^ (Fig. 1). Thiamin is produced from TMP by the action of TMP phosphatase (TH2), which can happen in the mitochondria or the cytosol^11,12^. In the cytosol, thiamin can be converted into the active form, thiamin diphosphate (TDP), by the action of pyrophosphokinase^13^ (Fig. 1).

Thiamin deficiency causes several health-related conditions from mild neurological and psychiatric symptoms (confusion, reduced memory, and sleep disturbances) to severe encephalopathy, ataxia, congestive heart failure and muscle atrophy^2^. Severe thiamin deficiency leads to a lethal disease known as beriberi, classically associated with diets that are low in thiamin and rich in carbohydrates^14^. In developing countries, thiamin deficiency remains widespread particularly due to high rates of white rice consumption^15,16^. Although progress has been made towards crop biofortification^17,18^, identifying production bottlenecks in the target pathway and/or understanding how pathways interact in the native metabolism is challenging^19,20^. Improving our knowledge on these factors prior to metabolic engineering can help to rationally improve production yields and crop nutritional values. Furthermore, the potential of gene editing to improve thiamin in rice grains is also under investigation^21^ and it requires a deep knowledge of the genes and the whole metabolic pathway. Thus, mathematical modeling is a powerful tool for gaining quantitative understanding on this pathway, since it allows to predict the metabolic effects of overexpression or knockout of specific genes, thereby allowing to rationally design metabolic engineering strategies^19^.

To build on the current understanding of thiamin biosynthesis in rice, we created a kinetic model of the thiamin pathway aiming to score the different potential biofortification strategies. Furthermore, we focused on *TH1* (HMP-P kinase/thiamin monophosphate synthase) to extend our knowledge on this gene which is crucial for thiamin biosynthesis. We strengthened the validation of its function by in silico analysis and yeast complementation assays. As a first test to evaluate the potential effect of *OsTH1* overexpression on thiamin content in rice endosperm, we overexpressed the gene in callus tissues, and confirmed the potential of *OsTH1* for metabolic engineering approaches aimed at enhancing vitamin B1 content through a cisgenic approach.

## Results

### *TH1* is a key gene for thiamin biofortification in rice

Aiming to identify key enzymes for thiamin biofortification, we developed a comprehensive kinetic mathematical model that simulates thiamin accumulation in *Oryza sativa*. The model encompasses 34 reactions, including external transport and inter-compartmental metabolite exchanges, and 29 metabolites (Tables S1 and S2). Given that thiamin is the primary B1 vitamer stored in rice seeds^1,20,28^, we focused on maximizing thiamin monophosphate (TMP) concentration, as stimulation of enzymes downstream of TMP was deemed unlikely to yield additional benefits to overall B1 levels in the endosperm tissue.

**Figure 2:**
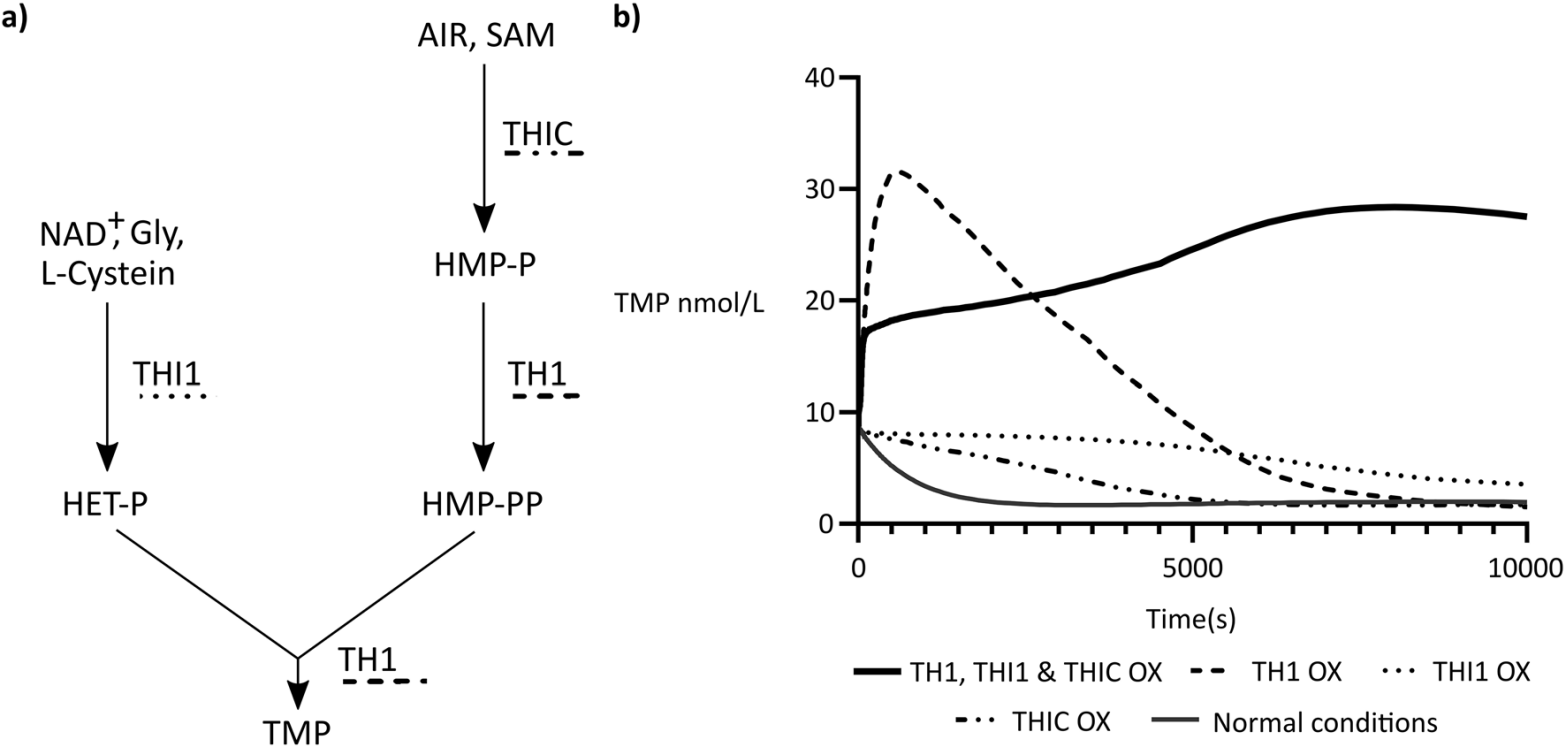
Simplified thiamin biosynthesis and model simulation results. (**a**) Simplified thiamin biosynthesis. THIC (4-amino-2-methyl-5-hydroxymethylpyrimidine phosphate (HMP-P) synthase) mediates the synthesis of HMP-P and THI1 (4-methyl-5-b-hydroxyethylthiazole phosphate (HET-P) synthase) of HET-P. HMP-P is phosphorylated into HMP-PP by the action of the bifunctional HMP-P kinase/TP synthase (TH1) which subsequently condenses HMP-PP and HET-P to form thiamin monophosphate (TMP). (**b**) Simulation results for TMP production along time in the non-optimized model (dark grey—Normal conditions; overexpression THIC: **−··−**; overexpression THI1: **····**; overexpression TH1: **– – –**; and overexpression TH1, THIC and THI1 simultaneously—black).

To emulate thiamin concentration in a steady state, we conducted time course simulations spanning 2 h and 47 min, allowing the formation of a plateau, using COPASI software. Under normal enzyme expression conditions, thiamin concentration plateaued at 1.97 nmol/L (Fig. 2). Subsequently, we systematically overexpressed the individual enzymes of the pathway by augmenting their Vmax by 100-fold. Enhancing THIC abundance yielded no discernible changes in TMP concentration, while augmenting THI1 abundance led to a modest increase in total TMP, from 1.97 to 3.56 nmol/L (1.8 fold; Fig. 2). Conversely, elevating TH1 abundance resulted in a TMP peak at 9 min 40 s (31.54 nmol/L); however, by the end of the time course, TMP levels reverted to those observed in the non-optimized model (Fig. 2).

We hypothesize that the increased flux towards THIC and THI1 fails to manifest the desired effect on TMP concentration due to the low turnover of HET-P and HMP-PP. Conversely, the rise in TH1 abundance, despite initially yielding elevated TMP concentration, fails to sustain the increase likely due to limited substrate availability. Consequently, we pursued a combinatorial approach by simultaneously increasing the abundance of all three enzymes. This resulted in a sustained rise in TMP over time—from 1.97 to 27.51 nmol/L (Fig. 2).

In light of these findings, we advocate for the simultaneous overexpression of *TH1*, *THI1*, and *THIC* as the most effective biofortification strategy for enhancing thiamin levels in rice endosperm. Particularly noteworthy is the role of TH1, where an increase in its abundance led to a transient peak in TMP concentration during early stages of the time course. Although this peak subsided by the end of the simulation, our results underscore the pivotal role of TH1 in driving thiamin biosynthesis.

### OsTH1 has all the conserved features of known thiamin monophosphate synthase/HMP-P kinase proteins

**Figure 3:**
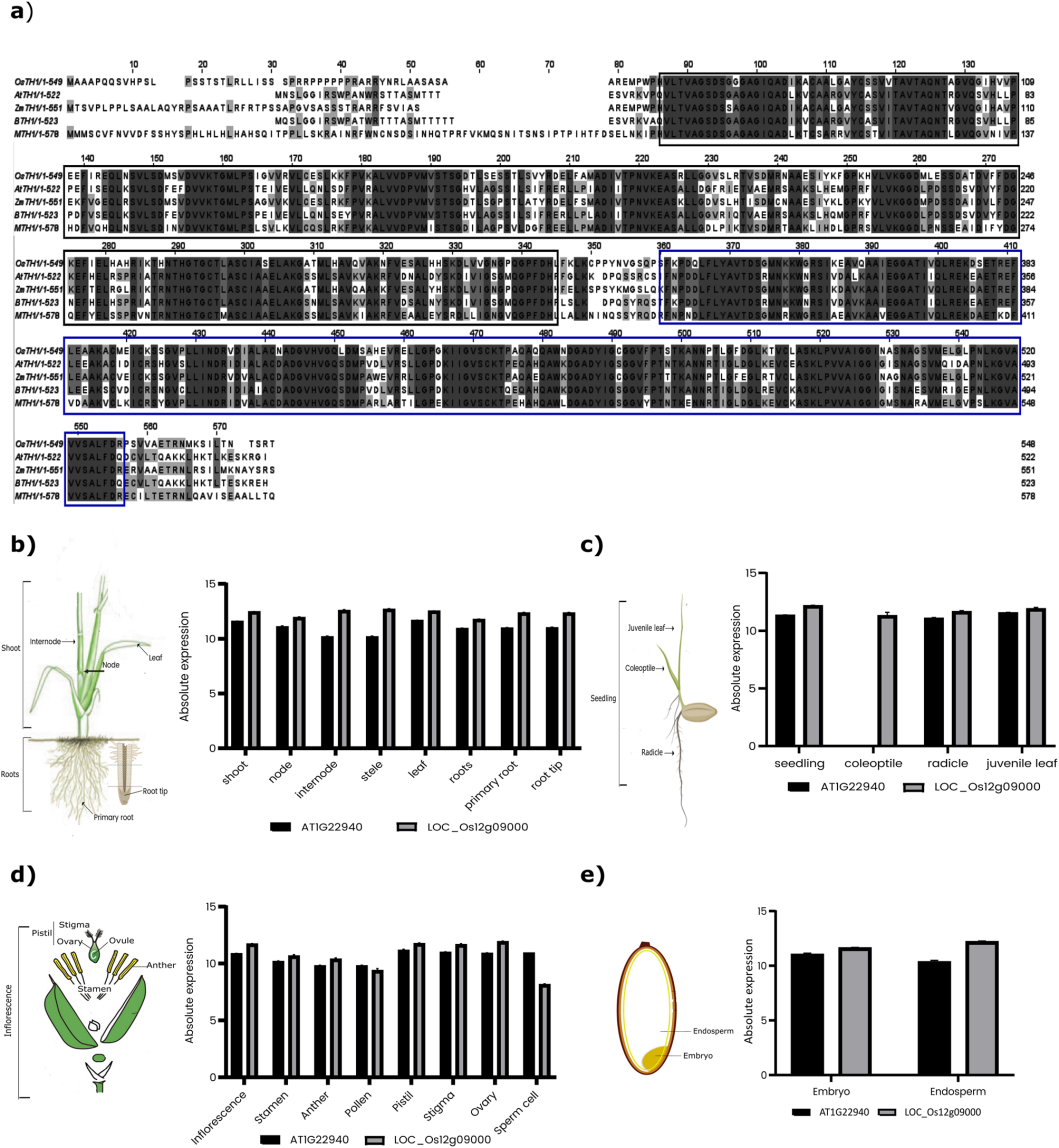
Comparative analysis of TH1 protein sequences and expression profile. (**a**) TH1 protein sequences of *Oryza sativa* (*OsTH1*), *Arabidopsis thaliana* (*AtTH1*), *Zea mays* (*ZmTH1*), *Brassica napus* (*BTH1*) and *Medicago truncatula* (*MTH1*). The alignment was performed using ClustalW and visualized in JalView. The black boxes are the conserved sequences of phosphomethylpyrimidine kinase domain and blue boxes the conserved sequences of thiamin monophosphate synthase domain. Expression profile in (**b**) shoot and root, (**c**) seedling, (**d**) inflorescence and (**e**) seed organs of *Arabidopsis* gene *TH1* (AT1522940) and *OsTH1* (LOC_Os12g09000), according to Genevestigator data. The absolute expression values shown represent expression values of the gene in a sample. Error bars represent differences in the biological replicates.

Recent studies have elucidated *OsTH1* (LOC_Os12g09000) role as a bifunctional enzyme involved in (1) the phosphorylation of HMP-P to HMP-PP and (2) in the condensation of HMP-PP and HET-P to yield TMP^10^ (Fig. 3). The coding sequence of *OsTH1* spans 548 amino acids and comprises two universally conserved domains across cellular organisms: HMP-P kinase domain from a.a. 33 to 291 and a thiamin monophosphate synthase (TMP synthase)/TenI domain from a.a. 313 to 513 according to Pfam and ScanProsite (Fig. 3a). Additionally, *OsTH1* features a putative N-terminal chloroplast transit peptide, consistent with other members of this plant protein family^29^.

In an effort to compare the gene expression profiles of *OsTH1* and Arabidopsis *TH1*, we used Genevestigator Database. We were able to verify that *OsTH1* shares an analogous expression profile with Arabidopsis *TH1* across diverse plant organs (Fig. 3b–e), thereby underscoring the functional convergence of these two genes.

### Phylogenetic analysis revealed that OsTH1 clusters with maize TH1 protein

**Figure 4:**
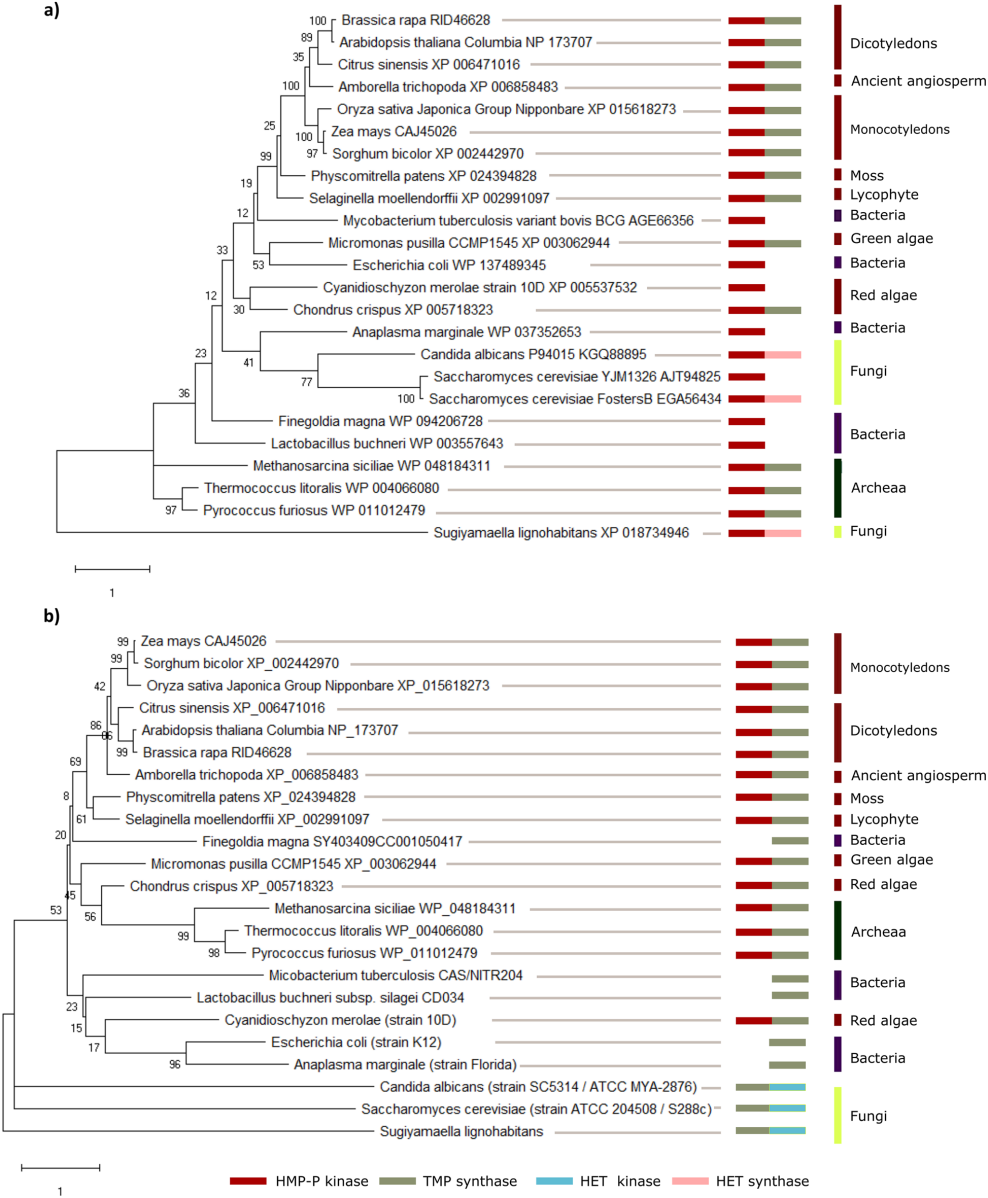
Phylogenetic analysis and domain composition of TH1 proteins. (**a**) Phylogenic tree developed with the sequences of the bifunctional TMP synthase/HMP-P kinase from plants and Archaea and the sequences of TMP synthase from microorganisms. (**b**) Phylogenic tree developed with the sequences of the bifunctional TMP synthase/HMP-P kinase from plants and Archaea and the sequences of HMP-P kinase from microorganisms. The numbers at the branching points indicate the percentage of times that each branch topology was found during bootstrap analysis (n = 1000). The boxes represent predicted functional domains: red—HMP-P kinase, grey—TMP synthase, blue—HET kinase and pink—HET synthase.

Phylogenetic trees were established using known and putative TH1 bifunctional proteins, encompassing representatives from Eubacteria (*Anaplasma marginale*,* Escherichia coli, Finegoldia magna*, *Lactobacillus buchneri*, *Mycobacterium tuberculosis*), Archaea (*Methanosarcina siciliae*, *Pyrococcus furiosus* and *Thermococcus litoralis*) and Eukarya (*Amborella trichopoda*, *Arabidopsis thaliana*, *Candida albicans*, *Brassica rapa*, *Citrus sinensis*, *Cyanidioschyson meroae*, *Micromonas pusilla*, *Physcomitrella patens*, *Saccharomyces cerevisiae*, *Selaginella moellendorffii*, *Sorghum bicolor, Sugiyamaella lignohabitans, Zea mays*), to ensure a comprehensive representation of the three kingdoms in the analysis. In bacteria and yeasts, TH1 activity is split between two separate enzymes (Fig. 4). Consequently, we developed two phylogenetic trees—one featuring TH1 proteins from plants and Archaea alongside TMP synthase proteins and the other with HMP-P kinase proteins from microorganisms.

As depicted in Fig. 4a,b, TH1 proteins from land plants formed a distinctive cluster in both phylogenetic trees. The conservation of HMP-P kinase/TMP synthase bifunctionality extends across algae and plant species, with both domains identified in the majority of analyzed sequences, except for *Cyanidioschyson meroae*. A parallel trend is evident in Archaea (Fig. 4a,b), where HMP-P kinase/TMP synthase bifunctionality also appears conserved. Conversely, members of Eubacteria exhibit only monofunctional enzymes. Interestingly, yeast TH1 displays distinct bifunctionalities, such as HMP-P kinase/HET synthase (Fig. 4a) and TMP synthase/HET kinase (Fig. 4b). *OsTH1* is closely related to bifunctional enzymes from plants, particularly showing a closer evolutionary relationship with maize *TH1*. This finding aligns with the documented close evolutionary ties between these species^30^ (Fig. 4a,b).

### *OsTH1* functionally complements yeast knock-out mutants

The functional validation of a gene of interest is often expedited through heterologous expression in yeast, serving as a rapid screening method^31^. Thus, we expressed *OsTH1* in yeast knockout mutants lacking thiamin monophosphate synthase and HMP-P kinase. In yeast, the bifunctional proteins *THI20* and *THI21*, encode HMP-P kinase, catalyzing the formation of HET in addition to their primary role^32,33^. Further, the condensation of HET-P and HMP-PP into TMP is catalyzed by the bifunctional TMP synthase/HET kinase enzyme encoded by the *THI6* gene^34^.

To delve into the role of *OsTH1* in vitamin B1 biosynthesis, we targeted *THI6* (strain Y01078) and *THI20/21*^32,35,36^ yeast mutants for heterologous expression. These mutants, deficient in thiamin monophosphate synthase and HMP-P kinase, require exogenous thiamin for growth. The complete cDNA of *OsTH1* was amplified and cloned into the yeast expression vector pPGK. *OsTH1* expression vector and the empty vector pPGK were transformed in *THI6*∆ and *THI20/21*∆. The wild type strain BY4741 of *S. cerevisiae* served as the positive control, while the yeast mutant strains transformed with the empty vector acted as negative controls. Complementation was confirmed by cell spotting assay and growth curve analysis.

**Figure 5:**
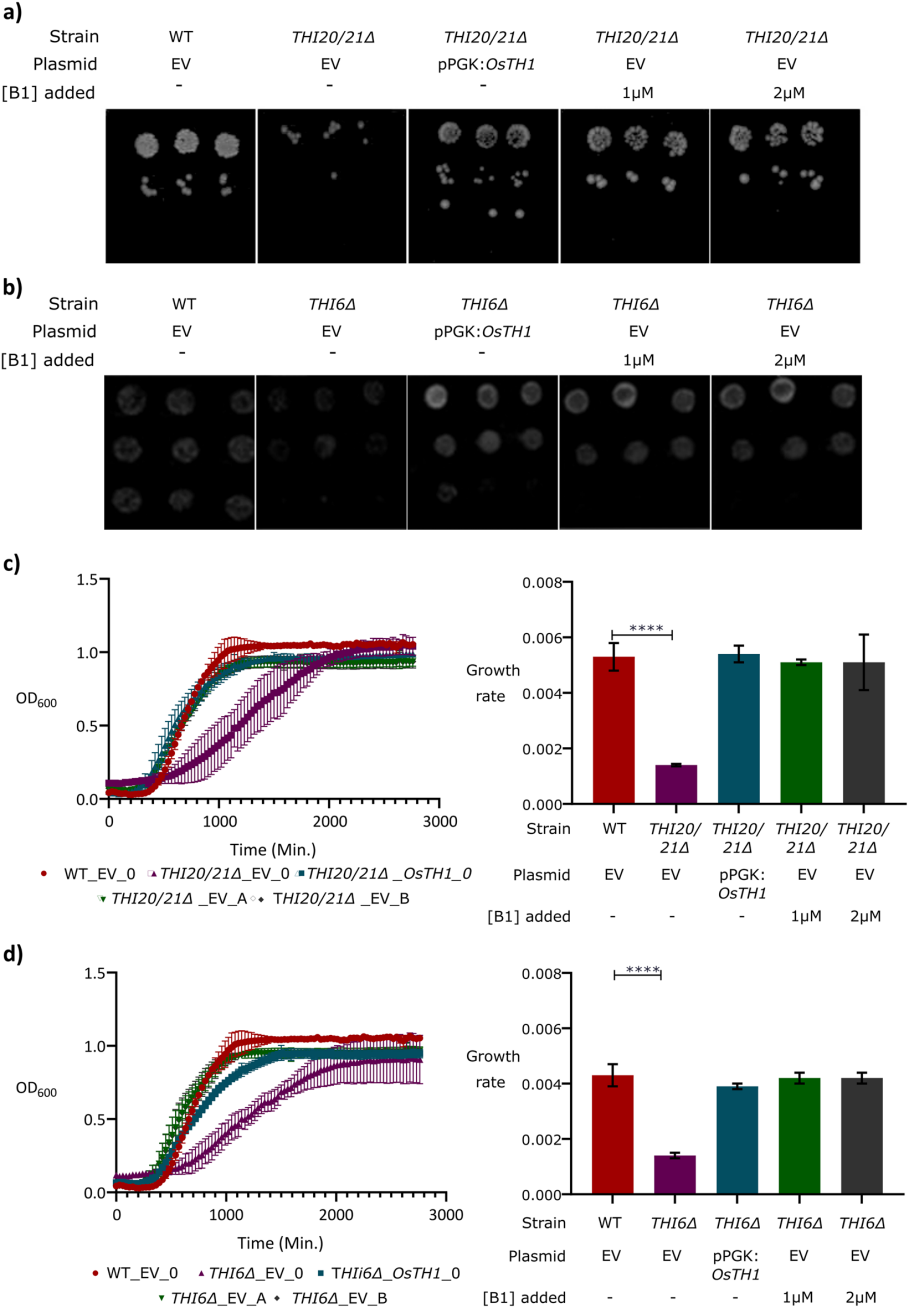
Functional complementation of *THI6∆* (deficient in thiamin monophosphate synthase) and *THI20/21∆* (deficient in HMP-P kinase) yeast mutants. The mutants transformed with the empty vector (EV) were used as negative control with no exogenous supply of thiamin (0) (*THI20/21∆_EV_0* and *THI6∆_EV_0)*, while the wild type transformed with the empty vector (EV) and no added thiamin (WT_EV_O) was used as positive control for all experiments. In all cases, *THI20/21∆*_*OsTH1*_0 and *THI6∆*_*OsTH1*_0 represent the mutants transformed with *OsTH1* with no added thiamin while *THI20/21∆*_EV_A, *THI6∆*_EV_A, *THI20/21∆*_EV_B and *THI6∆*_EV_B represent the complementation of the mutant with addition of 1μM thiamin (A) or 2 μM thiamin (B). (**a**) Cell spotting for *THI20/21∆*, deficient in HMP-kinase. (**b**) Cell spotting for *THI6∆*, deficient in TMP synthase. (**c**) Growth curve and growth rate of *THI20/21∆*, deficient in HMP-kinase. (**d**) Growth curve and growth rate of *THI6∆*, deficient in HMP-kinase. Data shown correspond to means ± standard errors of the means (SEM). Data was compared to control values (WT) by One-way ANOVA followed by Tukey’s post hoc test (****, *P* < 0.0001).

As illustrated in Fig. 5a,b, the expression of *OsTH1* restored the growth impairment of both *THI6∆* and *THI20/21∆* mutants on SD medium without thiamin. Moreover, when adding different thiamin concentrations (1 μM and 2 μM) to the medium, the growth was restored. Further confirmation of *OsTH1* complementation was achieved by monitoring yeast growth rate, which was consistent with the results from the cell spotting assay (Fig. 5c,d). *THI6∆* and *THI20/21∆* mutant strains transformed with the empty vector exhibited slower growth than the wild type, while those transformed with *OsTH1* reached the stationary phase before *THI6∆* and *THI20/21∆* mutants. These results provide robust support for the characterization *OsTH1* as a bifunctional enzyme, able to perform the activities of both thiamin monophosphate synthase and HMP-P kinase.

### *OsTH1* overexpression generates rice callus lines that accumulate thiamin

As a first approach to explore the potential applicability of the sole *OsTH1* overexpression for biofortification—a strategy hitherto unexplored in rice, despite its successful implementation in Arabidopsis^37^—we relied on rice callus. This is an optimal experimental platform due to its remarkable resemblance to the targeted endosperm tissue, underscored by the robust activity of endosperm-specific promoter within rice callus, despite their limited expression in other rice tissues^38^. Leveraging this unique feature, we incorporated *OsTH1* in a plant expression vector and stably transformed embryogenic calluses derived from mature seeds.

**Figure 6:**
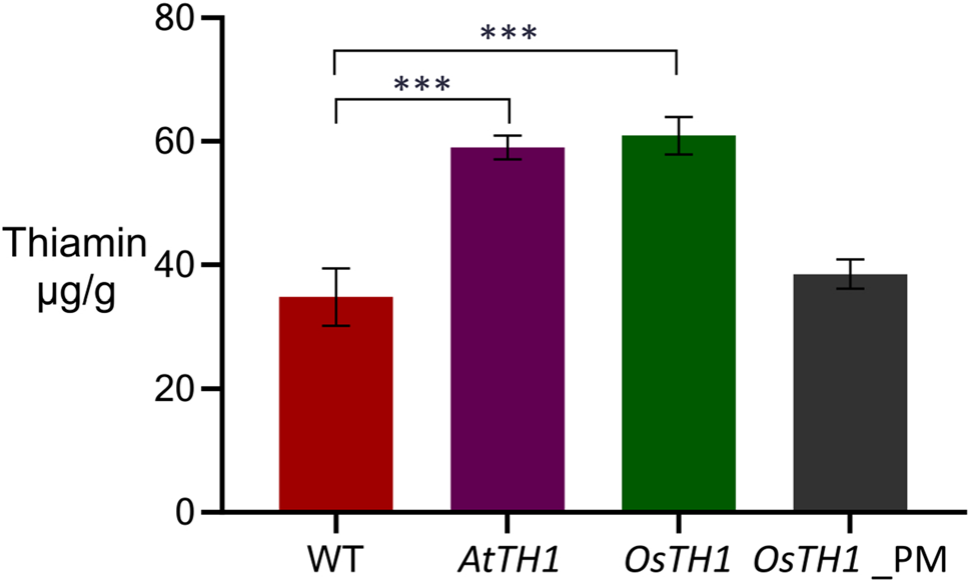
Thiamin content in rice callus, overexpressing *AtTH1* and *OsTH1*. *OsTH1* with a point mutation leading to an early stop codon was used to generate callus lines serving as negative control (*OsTH1_*PM). Twelve independent lines positive for each transgene were randomly pooled and stored at − 80 °C until thiamin extraction. Thiamin was detected and quantified by HPLC–MS. Data shown correspond to means ± standard errors of the means (SEM), N = 3. Data was compared to control values (WT) by One-way ANOVA followed by Tukey’s post hoc test (***, *P* < 0.001).

Rice callus were transformed with *OsTH1,* while *AtTH1* was used as positive control. For the negative control, we introduced an early stop codon in *OsTH1*. The callus expressing *OsTH1* showed a 50% increase in thiamin content (Fig. 6) compared with both wild type and the negative control. Specifically, the callus overexpressing *OsTH1* produced 60.9 µg of thiamin/g dry weight, providing the first evidence that *OsTH1* cisgenic approach indeed appears capable of significantly enhancing thiamin accumulation in transgenic callus.

## Discussion

The availability of a kinetic model for B1 pathway is fundamental to pinpoint the optimal candidates for implementing an accurate and knowledge-driven thiamin biofortification strategy. The development of this model was achieved by employing kinetic information in the form of ordinary differential equations, which enhances the accuracy of the predictions and facilitates the interpretation of the system behavior and sensitivity^39^. With parameters such as the Michaelis–Menten constant (Km) and Vmax (reaction rate when the enzyme is fully saturated by substrate), the model not only helps to identify the rate-limiting enzyme but also determines crucial enzymes for effective thiamin biofortification^40^. According to the model, achieving a sustained increase in B1 concentration requires not only improved HET-P and HMP-P production but also their condensation through the action of TH1. This is reasonably consistent with the literature, where an increased flux towards thiamin upon combined overexpression of *THIC*, *THI1* and *TH1*, enabled 2.5-fold enhancement in polished seeds^20^. However, it is vital to acknowledge the model’s assumptions, relying on standard rate laws and ideal solution conditions. Furthermore, our focus on de novo biosynthesis excludes considerations on intercellular trafficking, a factor that could affect thiamin content in specific tissues^17^. Although our model recognizes *OsTH1*, *OsTHI1* and *OsTHIC* combined expression as the best biofortification strategies, it also highlights TH1 as the single gene overexpression that leads to a significant change in thiamin concentration although not sustained over time. Based on this, we recommend undertaking the, not yet tried, exclusive overexpression of *TH1* in rice. Moreover, by making this model available, we aim to catalyze further advancements in the field of thiamin biofortification. Researchers can leverage and build upon our work, testing various scenarios and refining the model. In order to enhance the precision and reliability of our model, we encourage further studies focusing on the identification, validation of function, and kinetics of the enzymes involved in thiamin biosynthesis pathway. Investigating these aspects will not only contribute to a more comprehensive understanding of thiamin metabolism in rice but will also provide crucial data for refining the model.

Recognizing the pivotal role of *TH1* in B1 biosynthesis, we delved deeper into its analysis and showed that (1) it has all the conserved features of known thiamin phosphate synthase/HMP-P kinase proteins, (2) it has the same expression profile as TH1 described for Arabidopsis, (3) in the phylogenetic analysis it formed a cluster with the known enzyme from *Zea mays* and (4) it functionally complements the yeast knock out thiamin monophosphate synthase and HMP-P kinase mutants, supporting its established role as TMP synthase/HMP-P kinase.

TH1 proteins and ubiquitous across different life forms and our phylogenetic analysis of TH1 proteins provided insights into their evolutionary history. Apart from the monofunctional enzymes found in bacteria, our findings reveal three distinct bifunctionalities: HMP-P kinase/TMP synthase, adopted by plants and Archaea, TMP synthase/HET kinase and HMP-P kinase/HET synthase. Both adopted by fungi. The HMP-P kinase/TMP synthase bifunctionality present in plants and Archaea facilitates the coordinated activity of the two enzymes in the biosynthetic pathway, given that the product of HMP-P kinase (HMP-PP) serves as the substrate for TMP synthase (Fig. 1)^3,10^. This arrangement facilitates the efficient exchange of HMP-PP between the two reaction sites, effectively channeling metabolites through the biosynthetic pathway. Both Archaea and plants seem to have adopted the same strategy to minimize the premature release of HMP-PP before its condensation with HET-P. In contrast, yeasts employ a different approach, with TMP synthase/HET kinase and HMP-P kinase/HET synthase bifunctionalities (Fig. 4a,b). In the former case, the strategy mirrors that of plants and Archaea by minimizing the release of HET-P prior to its condensation with HMP-PP by TMP synthase. In the latter case, enzyme dual functions involve the formation of the thiazole and pyrimidine moieties (Fig. 1), representing the initial steps of vitamin B1 metabolic pathway. This arrangement might ensure the presence of both precursors for the correct flux of metabolites during thiamin biosynthesis. *OsTH1* is closely related to bifunctional enzymes from plants, especially sharing a closer evolutionary relationship with maize *TH1*.

Our results collectively affirm the functionality of *OsTH1* as well as its utility in metabolic engineering approaches aimed at enhancing vitamin B1 content in rice. Such an insight into the endogenous thiamin metabolism of rice is particularly pertinent for biofortification efforts, where extensive knowledge of key biosynthetic genes is essential. The proven functionality of rice *TH1* supports its application in metabolic engineering through a cisgenic approach, aligning with positive consumer perceptions^41^. In rice callus, the sole overexpression of *OsTH1* effectively redirected the thiamin biosynthetic equilibrium, resulting in a substantial increase in vitamin B1 accumulation. While the kinetic model predicted that the increase resulting from *TH1* overexpression would not be sustained over time, the rice callus experiments demonstrated a maintained elevation. Nonetheless, and acknowledging the imperfections due to the necessity for additional kinetic studies on thiamin biosynthetic enzymes and regulation, our model played a crucial role in identifying *TH1* as a reliable target. These findings underscore the potential of *OsTH1* overexpression as a promising strategy for thiamin biofortification in rice, shedding light on its unique metabolic engineering capabilities, hitherto unexplored in this essential crop.

## Methods

### Kinetic model construction and parameter selection

The metabolism of thiamin in plants is described^6,37,42^ (Fig. 1). Based on this biochemical knowledge and on kinetic constants available on the literature^29,43–47^ (Table S2), a model could be derived. The metabolic network of vitamin B1 in rice was constructed in a stepwise manner, by integrating genomic data gathered from MSU Rice Genome Annotation Project^48^ and National Center for Biotechnology Information (NCBI)^49^, and biochemical data through manual curation of an extensive survey of scientific literature^13,29,43–47,50–56^ and public databases such as KEGG^57^, MetaCyc^58^, Uniprot^59^ and BRENDA^60^. Only the reactions directly involved in the pathways were included in the model (Table S1). In cases where the kinetic parameters were not available in the literature, the stoichiometric model of vitamin B1 metabolism (Tables S3 and S4, and Fig. S1) was used to predict the reaction flux. Time course simulations were performed to assess TMP production over time, using the deterministic model (LSODA) from COPASI^61^, with a duration of 10,000 s (2 h and 47 min) to simulate the formation of a plateau.

A simple Michaelis–Menten type reaction was assumed for most of the enzyme kinetics^62^. When appropriate (TH1, THIC and THiM reactions, Table S2), the kinetic equation for a two-substrate reaction was also modeled^63^. These expressions were used in a set of ordinary differential equations to account for the time dependence of the metabolite concentration. The initial concentration of the metabolites was decided based on the literature^20,28,64–66^ (Table S2). All reactions and respective stoichiometry as well as the respective kinetic parameters are presented on Tables S1 and S2.

### OsTH1 sequence and phylogenetic analysis

The gene expression data of the putative OsTH1 was compared with the one from Arabidopsis by analyzing the data available at Genevestigator (NEBION)^67^ from different stages of plant development. For the phylogenetic analysis, the full-length protein sequence from *Arabidopsis thaliana TH1* (AT1G22940) was used as a query to run Blastp in order to identify orthologs of *TH1* gene. The presence of functional HMP-P kinase and thiamin monophosphate (TMP) synthase domains was evaluated in the putative ortholog protein sequences using Pfam^68^ and ScanProsite^69^. The full-length amino acid sequences were aligned in MEGA X^70^ using MUSCLE software^71^. The WAG model was selected using SMS (Smart Model Select)^72^ and the phylogenetic relationship inferred using the maximum likelihood method. The maximum likelihood tree was evaluated with 1000 bootstrap replicates. Two phylogenetic trees were developed: (1) with the bifunctional TMP synthase/HMP-P kinase proteins from plants and Archaea and with the TMP synthase from microorganisms and (2) with the bifunctional TMP synthase/HMP-P kinase protein from plants and Archaea and with the HMP-P kinase from microorganisms. Phylogenetic trees were visualized using FigTree v1.4.2.

### OsTH1 cloning

Based on the predicted DNA sequences of *OsTH1* from MSU Rice Genome Annotation Project, 3′ and 5′ primers were designed (Table S5) and the coding sequence was amplified from *Oryza sativa* japonica cv. Nipponbare cDNA. The DNA fragment was purified using High Pure PCR Product Purification Kit (Roche) and was subsequently cloned into the pJET 1.2 vector (Thermo Scientific CloneJET PCR Cloning Kit).

For yeast complementation assays, the pPGK-*OsTH1* expression vector was generated by Gibson assembly (Gibson assembly master mix, New England Biolabs). *OsTH1* was amplified from pJET1.2-*OsTH1* vector while pPGK was amplified (Table S5) in order to create complementary overhangs. The sequences were verified by DNA sequencing.

For rice transformation, the expression clones were generated by Gateway cloning. *OsTH1* was amplified from pJET1.2-*OsTH1* primer pair and cloned into pDONR221 (Invitrogen, CA, United States) according to manufacturer’s instructions. This vector was then cloned into Multisite Gateway vector pHb7m24GW, 3 to yield pHb-ZmUbi1-OsTH1-t35S^73^. As negative control, an early stop codon was introduced in *OsTH1* using QuickChange II Site-directed mutagenesis kit (Agilent technologies, EUA). Introduction of the point mutation was confirmed by sequencing and the sequence cloned into Multisite Gateway vector pHb7m24GW, 3. As positive control, Arabidopsis *TH1* was amplified from cDNA, cloned into pDONR221 followed by the same cloning strategy as OsTH1 to yield pHb-ZmUbi1-AtTH1-t35S. The final vectors prepared were analyzed by digestion with restriction enzymes.

The *E. coli* DH5α strain grown in LB medium (supplemented with the appropriate antibiotics) was used for bacterial transformation.

### Yeast strains, culture conditions, transformation, and complementation assay

The yeast strains used in this study were the following: BY4741 (MATa his3Δ1 leu2Δ0 met15Δ0 ura3Δ0), Y01078 (BY4741; MATa; ura3Δ0; leu2Δ0; his3Δ1; met15Δ0; YPL214c::kanMX4), CEN.FE23-5A (CEN.PK; MATa; ura3-52; leu2-3,112; his3-Δ1; YOL055c::URA3) (EUROSCARF) and CEN.FE23-5A THI21 (CEN.PK; MATa; ura3-52; leu2-3,112; his3-Δ1; YOL055c::URA3 THI21 Δ::HIS3)^36^. Yeast strains were grown in YPD medium (10 g/L yeast extract, 20 g/L peptone, 20 g/L D-glucose) and in synthetic medium (20 g/L glucose, 6.7 g/L YNB and 19 g/L agar, arginine 20 mg/L, isoleucine 30 mg/L, lysine 30 mg/L, methionine 20 mg/L, phenylalanine 50 mg/L, threonine 200 mg/L, tyrosine 200 mg/L, valine 150 mg/L, adenine 100 mg/L, leucine 100 mg/L) lacking specific amino acids (SD medium). The preparation of yeast competent cells and transformation were performed by the Lithium-Acetate (LiAc) method^74^. The mutants transformed with the pPGK empty vector were use as negative control while the wild type transformed with the empty pPGK was used as positive control. Phenotypic growth assays were carried out by spotting 5 μL of early exponential phase cultures (OD_600_ 0.4) and sequentially diluted (1/10 and 1/100) in SD medium without thiamin and plated in SD medium (lacking uracyl) containing 0 μM, 1 μM or 2 μM of thiamin HCL. The plates were incubated for 3 days at 30 °C, after which the colonies were photographed. For the growth curves, growth was monitored by OD_600_ measurements, starting at an OD_600_ of 0.05, every 30 min under continuous shaking in the Automated Microbiology Growth Analysis System (Bioscreen C) for 3 days at 30 °C. These assays were independently repeated four times.

### Plant material and transformation

The binary vectors described earlier were introduced separately in *Agrobacterium tumefaciens* strain EHA105 and transformed into embryogenic callus developed from mature seeds of rice (*Oryza sativa* japonica cv. Nipponbare) following established methods^75^. Calluses were maintained in embryogenic induction medium (EIM)^76^ for one month to develop highly embryogenic callus with a large number of somatic embryos. To verify transgene integration, the hygromycin gene was amplified. Twelve independent lines positive for each transgene were randomly pooled, freeze dried and stored at − 80 °C.

### Thiamin extraction and quantification

The homogenized samples (100 mg each) were extracted with 1 ml of 50 mM of phosphate buffer containing thiamin-d_3_ as internal standard. The extract was vortexed for 10 s and stored for 2 h at 4 °C. Then, it was purified with an Amicon 3KDa centrifugal filter (15 900 rcf at 4°C for 20 min) before loading to a liquid chromatography coupled with tandem mass spectrometry system (LC–MS). The LC–MS was a Waters ACQUITY UPLC and an Applied Biosystems API 4 000 MS equipped with an electrospray ionization source. The extracted thiamin was separated on a Waters ACQUITY UPLC HSS T3 Column (2.1 mm × 150 mm, 100 Å, 1.8 µm) equipped with a Waters ACQUITY UPLC HSS T3 VanGuard Pre-column (2.1 mm × 5 mm, 100 Å, 1.8 µm) maintained at 45 °C.

### Statistical analysis

To compare the growth of the yeast mutants and wild type, the growth rate of each culture was calculated after data transformation to natural log-values and selection of the exponential phase. A non-linear regression was applied, and the slope of the given curve used to compare the different groups using One-way ANOVA followed by Tukey’s post hoc test. To compare the concentration of thiamin in different callus lines One-way ANOVA followed by Tukey’s post hoc test was used. Significant differences in means, are indicated for datasets in which *P* values below 0.05 are considered significant, and P-values below 0.01 are considered very significant. The statistical analysis was performed using GraphPad8.

### Supplementary Information

Supplementary Information.

## Data Availability

The datasets supporting the conclusions of this article are included within the article and its additional files. The sequence of LOC_Os12g09000 is available at NCBI, as accession number NC_029267.1.
