# Supplementary material for: OsTH1 is a key player in thiamin biosynthesis in rice
Source: Sci Rep. 2024 Jun 12;14:13591. doi: 10.1038/s41598-024-62326-2 (PMC11169455; doi:10.1038/s41598-024-62326-2)
Supplement: Supplementary file 1 — Supplementary Information. [file 41598_2024_62326_MOESM1_ESM.pdf]

Supplementary Material

OsTH1 is a Key Player in Thiamin Biosynthesis in Rice

Maria Faustino[1,2], Tiago Lourenço[1], Simon Strobbe[2,a], Da Cao[2], André Fonseca[1], Isabel Rocha[1], Dominique Van Der Straeten[2,*], M. Margarida Oliveira[1,*]

[1]Instituto de Tecnologia Química e Biológica António Xavier, Universidade Nova de Lisboa, 2780-157, Oeiras, Portugal.
[2]Laboratory of Functional Plant Biology, Department of Biology, Ghent University, K. L. Ledeganckstraat 35, B-9000 Gent, Belgium.
[a]Current address: University of Geneva, Quai E. Ansermet 30, 1211 Geneva, Switzerland.
*dominique.vanderstraeten@ugent.be; mmolive@itqb.unl.pt

Supporting Tables

Table S1. Detailed list of kinetic model reactions.

**THI1**	NAD_p + Gly_p + LCys_p → HET-P_p + NA	**(1)**
**TH1B**	HMP-PP_p + HET-P_p → TMP_p	**(2)**
**THIC**	AIR_p + SAM → HMP-P_p	**(3)**
**TH1A**	HMP-P_p + ATP_p → ADP_p + HMP-PP_p	**(4)**
**TH1A_rv**	HMP_p + ATP_p → ADP_p + HMP-P_p	**(5)**
**TH2_p**	TMP_p → Thiamin_p	**(6)**
**TMP transport from plastid to cytosol**	TMP_p → TMP_c	**(7)**
**Thiamin transport from plastid to cytosol**	Thiamin_p → Thiamin_c	**(8)**
**TH2_c**	TMP_c → Thiamin_c	**(9)**
**TMP transport from cytosol to mitochondria**	TMP_c → TMP_m	**(10)**
**Thiamin transport from plastid to cytosol**	Thiamin_c → Thiamin_m	**(11)**
**TPK_c**	Thiamin_c → TDP_c	**(12)**
**Transport of TDP from cytosol to plastid**	TDP_c → TDP_p	**(13)**
**Transport of TDP from cytosol to mitochondria**	TDP_c → TDP_m	**(14)**
**TH2_m**	TMP_m → Thiamin_m	**(15)**
**Transport of TDP from cytosol to peroxisome**	TDP_c → TDP_px	**(16)**
**ThiM**	HET_c + ATP_c → HET-P_c + ADP_c	**(17)**
**Transport of HET-P from cytosol to plastid**	HET-P_c → HET-P_p	**(18)**

**Import of HET**	→ HET_c	**(19)**
**Import of HMP**	→ HMP_p	**(20)**
**Input of thiamin**	Thiamin_out → Thiamin_c	**(21)**
**Export of TDP_m**	TDP_m →	**(22)**
**Export of TDP_px**	TDP_px →	**(23)**
**Export of TDP_p**	TDP_p →	**(24)**
**Import of SAM**	→ SAM	**(25)**
**Import of NAD**	→NAD_p	**(26)**
**Import of glycine**	→ Gly_p	**(27)**
**Import of cysteine**	→ LCys_p	**(28)**
**Import of ATP**	→ ATP_c	**(29)**
**Export of ADP**	ADP_c →	**(30)**
**Export of NA**	NA →	**(31)**
**Export of thiamin**	→ Thiamin_out	**(32)**
**Import of AIR**	→ AIR_p	**(33)**
**Thiamin transport from mitochondria to cytosol**	Thiamin_m → Thiamin_c	**(34)**

Abbreviations: **Metabolites: ADP**: adenosine diphosphate; **AIR**: 5-aminoimidazole ribotide; **ATP**: adenosine triphosphate; **HET:** 4-methyl-5-hydroxymethylthiazole; **Gly**: glycine; **HET-P**: 4-methyl-5-hydroxymethylthiazole phosphate; **HMP-P**: 4-amino-2-methyl-5-hydroxymethylpyrimidine phosphate; **HMP-PP**: 4-amino-2--methyl-5-hydroxymethylpyrimidine diphosphate; **LCys:** L-cysteine; **NA**: nicotinic acid; **NAD**: nicotinamide adenine dinucleotide; **SAM:** S-adenosyl methionine**; TDP**: thiamin diphosphate; **TMP**: thiamin monophosphate. **Compartments**: **_p**: plastid; **_c**: cytosol; **_m**: mitochondria; **_px:** peroxisome. **Reactions: THI1** - thiazole biosynthetic enzyme; **TH1B** - thiamin-phosphate synthase; **THIC** - hydroxymethylpyrimidine phosphate synthase; **TH1A** - hydroxymethylpyrimidine kinase; **TH1A_rev –** Reverse activity of hydroxymethylpyrimidine kinase; **TH2** - thiamin monophosphate phosphatase; **TPK** - thiamin pyrophosphokinase; **ThiM -** Hydroxyethylthiazole kinase.

Table S2. Rate law (RL) equations, kinetic parameters, and respective references for each reaction of included in the kinetic model.

Reac. Nr	Reaction	E. C. number	Equation	Parameters	Reference
**1**	THI1	2.4.2.60	Flux4	Flux= 1.7	-
**2**	TH1B	2.5.1.3	$\frac{V_{max} \times A \times B}{K_{mA} \times K_{mB} + A \times K_{mB} + B \times K_{mA} + A \times B}$	V_{max}= 8.5<br>K_{mA}= 1790<br>K_{mB}= 12800	[1]
**3**	THIC	4.1.99.17	$\frac{V_{max} \times A \times B}{K_{mA} \times K_{mB} + A \times K_{mB} + B \times K_{mA} + A \times B}$	V_{max}=0.1128<br>K_{mA} =1700<br>K_{mB} =17000	[2]
**4**	TH1A	2.7.1.49	$\frac{V_{max} \times A \times B}{K_{mA} \times K_{mB} + A \times K_{mB} + B \times K_{mA} + A \times B}$	V_{max} =20.1<br>K_{mA} =7240<br>K_{mB} =6580	[1]
**5**	TH1A_rev	2.7.1.49	$\frac{V_{max} \times A \times B}{K_{mA} \times K_{mB} + A \times K_{mB} + B \times K_{mA} + A \times B}$	V_{max} =20.1<br>K_{mA} =7240<br>K_{mB} =6580	[1]
**6**	TH2_p	3.1.3.100	$\frac{V_{max} \times A}{K_m + A}$	V_{max} =60<br>K_m =49000	[3]
**7**	TMP transport from plastid to cytosol	-	Flux 1	Flux= 1.7	-
**8**	Thiamin transport from plastid to cytosol	-	Flux2	Flux=0.374	-
**9**	TH2_c	3.1.3.100	$\frac{V_{max} \times A}{K_m + A}$	V_{max} =60<br>K_m =49000	[3]
**10**	TMP transport from cytosol to mitochondria	-	Flux 1	Flux= 1.7	-
**11**	Thiamin transport from plastid to cytosol	-	Flux2	Flux=0.374	-
**12**	TPK	2.7.6.2	$\frac{V_{max} \times A}{K_m + A}$	V_{max} =0.374<br>K_m =13000	[3]
**13**	Transport of TDP from	-	Flux2	Flux=0.374	-

	cytosol to plastid				
**14**	Transport of TDP from cytosol to mitochondria	-	Flux2	Flux=0.374	-
**15**	TH2_m	3.1.3.100	$\frac{V_{max} \times A}{K_m + A}$	V_{max} =60<br>K_m =49000	[3]
**16**	Transport of TDP from cytosol to peroxisome	-	Flux2	Flux=0.374	-
**17**	ThiM	2.7.1.50	$\frac{V_{max} \times A \times B}{K_{mA} \times K_{mB} + A \times K_{mB} + B \times K_{mA} + A \times B}$	V_{max}=0.4302<br>K_{mA} =17500<br>K_{mB} =21800	[4]
**18**	Transport of HET-P from cytosol to plastid	-	Flux3	Flux=0.01	-
**19**	Import of HET	-	Flux3	Flux=0.01	-
**20**	Import of HMP	-	Flux2	Flux=0.374	-
**21**	Import of thiamin	-	$\frac{V_{max} \times A}{K_m + A}$	V_{max}=0.1693<br>K_m =43300	[5]
**22**	Export of TDP_m	-	Flux2	Flux=0.374	-
**23**	Export of TDP_px	-	Flux2	Flux=0.374	-
**24**	Export of TDP_p	-	Flux2	Flux=0.374	-
**25**	Import of SAM	-	Mass action	V_{max}=5.95	[6]
**26**	Import of NAD	-	Flux2	Flux=0.374	-
**27**	Import of Glycin	-	Flux2	Flux=0.374	-
**28**	Import of Cysteine	-	Flux2	Flux=0.374	-
**29**	Import of ATP	-	Flux2	Flux=0.374	-
**30**	Export of ADP	-	Flux2	Flux=0.374	-
**31**	Export of NA	-	Flux2	Flux=0.374	-
**32**	Export of thiamin	-	Flux1	Flux1=1.7	-

**33**	Import of AIR	-	Flux2	Flux=0.374	-
**34**	Thiamin transport from mitochondria to cytosol	-	Flux2	Flux=0.374	-

Abbreviations: **THI1** - thiazole biosynthetic enzyme; **TH1B** - thiamin-phosphate synthase; **THIC** - hydroxymethylpyrimidine phosphate synthase; **TH1A** - hydroxymethylpyrimidine kinase; **TH1A_rev –** Reverse activity of hydroxymethylpyrimidine kinase; **TH2** - thiamin monophosphate phosphatase; **TPK** - thiamin pyrophosphokinase; **ThiM -** Hydroxyethylthiazole kinase. In cases where the kinetic parameters were not available in the literature, the stoichiometric model of vitamin B1 metabolism was used to predict the reaction flux. **AIR**: 5-aminoimidazole ribotide; **HET:** 4-methyl-5-hydroxymethylthiazole; **HET-P**: 4-methyl-5-hydroxymethylthiazole phosphate; **HMP-P**: 4-amino-2-methyl-5-hydroxymethylpyrimidine phosphate; **NA**: nicotinic acid; **NAD**: nicotinamide adenine dinucleotide; **SAM:** S-adenosyl methionine**; TDP**: thiamin diphosphate; **TMP**: thiamin monophosphate. **Compartments**: **_p**: plastid; **_c**: cytosol; **_m**: mitochondria; **_px:** peroxisome.

Supporting table S3. Set of reactions included in the reconstructed pathway for the stoichiometric model.

Nr	Reaction	EC	co	Equation
**R1**	THIC -Hydroxymethylpyrimidine phosphate synthase	4.1.99.17	p	$AIRp \rightarrow HMPP\_p$
**R2**	TH1A - Hydroxymethylpyrimidine kinase	2.7.1.49	p	$HMPP\_p \rightarrow HMPPP\_p$
**R3**	THI1 - Thiazole biosynthetic enzyme	2.4.2.60	p	$NAD\_p \rightarrow HETP\_p$
**R4**	TH1B - Thiamin-phosphate synthase	2.5.1.3	p	$HETP_p + HMPPP\_p \rightarrow TMP\_p$
**R5**	TMP transport from plastid to cytoplasm	-	c	$TMP\_p \rightarrow TMP\_c$
**R6**	TH2A - Thiamin monophosphate phosphatase	3.1.3.100	c	$ThMP\_c \rightarrow Thiamin\_c$
**R7**	TPK - Thiamin pyrophosphokinase	2.7.6.2	c	$Thiamin\_c \rightarrow ThDP\_c$
**R8**	TDP transport from cytoplasm to plastid	-	c	$TDP\_c \rightarrow TDP\_p$
**R9**	TMP transport from cytoplasm to mitochondria	-	m	$TMP\_c \rightarrow TMP\_m$
**R10**	TH2B - Thiamin monophosphate synthase	-	m	$TMP\_m \rightarrow Thiamin\_m$
**R11**	Thiamin transport from mitochondria to cytoplasm	-	c	$Thiamin\_m \rightarrow Thiamin\_c$
**R12**	TDP transport from cytoplasm to mitochondria	-	m	$TDP\_c \rightarrow TDP\_m$
**R13**	Transport of AIR into the plastid	-	p	$AIR\_out \rightarrow AIR\_p$
**R14**	Transport of TDP out of the cytoplasm	-	c	$TDP\_c \rightarrow TDP\_out$
**R15**	Transport of TMP out of the mitochondria	-	c	$TMP\_c \rightarrow TMP\_out$
**R16**	Transport of NAD into the cytoplasm	-	c	$NADP\_out \rightarrow NADP\_p$

Abbreviations: **Co:** compartment; **p**: plastid; **c**: cytosol; **m**: mitochondria.

Supporting table S4. Stoichiometric matrix used in the development of the stoichiometric model.

	r1	r2	r3	r4	r5	r6	r7	r8	r9	r10	r11	r12	r13	r14	r15	r16
**AIR_p**	-1	0	0	0	0	0	0	0	0	0	0	0	1	0	0	0
**HMPP_p**	1	-1	0	0	0	0	0	0	0	0	0	0	0	0	0	0
**HMPPP_p**	0	1	0	-1	0	0	0	0	0	0	0	0	0	0	0	0
**NAD_p**	0	0	-1	0	0	0	0	0	0	0	0	0	0	0	0	1
**HETP_p**	0	0	1	-1	0	0	0	0	0	0	0	0	0	0	0	0
**TMP_p**	0	0	0	1	-1	0	0	0	0	0	0	0	0	0	0	0
**TMP_c**	0	0	0	0	1	-1	0	0	-1	0	0	0	0	0	0	0
**Thiamin_c**	0	0	0	0	0	1	-1	0	0	0	1	0	0	0	0	0
**TDP_c**	0	0	0	0	0	0	1	-1	0	0	0	-1	0	-1	0	0
**TDP_p**	0	0	0	0	0	0	0	1	0	0	0	0	0	0	0	0
**TDP_m**	0	0	0	0	0	0	0	0	1	-1	0	0	0	0	-1	0
**Thiamin_m**	0	0	0	0	0	0	0	0	0	1	-1	0	0	0	0	0
**ThDP_m**	0	0	0	0	0	0	0	0	0	0	0	1	0	0	0	0

Abbreviations: **AIR_p**: 5-aminoimidazole ribotide; **HMPP_p**: 4-amino-2-methyl-5-hydroxymethylpyrimidine phosphate; **HMPPP_p**: 4-amino-2--methyl-5-hydroxymethylpyrimidine diphosphate; **NAD_p**: nicotinamide adenine dinucleotide; **HETP_p**: 4-methyl-5-hydroxymethylthiazole phosphate; **TMP_p**: thiamin monophosphate; **TMP_c**: thiamin monophosphate; **Thiamin_c**: thiamin; **TDP_c**: thiamin diphosphate; **TDP_p**: thiamin diphosphate; **TDP_m**: thiamine diphosphate; **Thiamin_m**: thiamin; **TDP_m**: thiamin diphosphate; **R1**: THIC -hydroxymethylpyrimidine phosphate synthase; **R2**: TH1A - hydroxymethylpyrimidine kinase; **R3**: THI1 - thiazole biosynthetic enzyme; **R4**: TH1B - thiamin-phosphate synthase; **R5**: trans_TMP_p_c - TMP transport from plastid to cytoplasm; **R6**: TH2A - thiamin monophosphate phosphatase; **R7**: TPK - thiamin pyrophosphokinase; **R8**: Tr_TDP_c_p - TDP transport from cytoplasm to plastid; **R9**: Tr_TMP_c_m - TMP transport from cytoplasm to mitochondria; **R10**: TH2B - thiamin monophosphate phosphatase B; **R11**: Tr_Thiamin_m_c - thiamin transport from mitochondria to cytoplasm; **R12**: Tr_TDP_c_m - TDP transport from cytoplasm to mitochondria; **R13**: Tr_in_AIR_p - transport of AIR into the plastid; **R14**: Tr_TDP_out_c - transport of TDP out of the cytoplasm; **R15**: Tr_TMP_out_m - transport of TMP out of the mitochondria; **R16**: Tr_NAD_in_c - transport of NAD into the cytoplasm.

Supporting table S5. Primers used in the study.

Primer pair	Sequence 5' → 3'	Use
**1**	ATGGCGGCCGCACCACAG	*OsTH1* amplification from cDNA
	CTAGGTTCTAGAGGTGTTGG	
**2**	ACAACAAATATAAAACACCCATGGCGGCCGCACCACAG	*OsTH1* amplification for Gibson assembly
	TCAATTCAATTCAATGGATCCTAGGTTCTAGAGGTG TTGGTCAAGATGGATTTC	
**3**	GATCCATTGAATTGAATTGAAATC	

	GGGTGTTTTATATTTGTTGTAAAAAG	Ppgk amplification for Gibson assembly
**4**	GGGGACAAGTTTGTACAAAAAAGCAGGCTTAATGGCGGCCGCACCACAG	*OsTH1* amplification for gateway cloning
	GGGGACCACTTTGTACAAGAAGCTGGGTACTAGGTTCTAGAGGTGTTGGTCAAGATG	
**5**	GGATGAACTGACTATTGGGGAGCAGCCGC	*OsTH1* point mutation
	GCGGCTGCTCCCCAATAGTCACTTCATCC	
**6**	GGGGACAAGTTTGTACAAAAAAGCAGGCTTAATGAATAGCTTAGGAGGAATTAGG	*AtTH1* amplification from cDNA
	GGGGACCACTTTGTACAAGAAGCTGGGTATCAAATTCCCCTTTTGCTC	
**7**	TCTTGCCACAATGACCTATTCTACAGTTAATATTAATACATCGTACGCTGCAGGTC	*THI21* deletion from yeast genome
	ATCGATGTGTCTATTCATTCAT ATTCTGAGCGGCGGTCCTAGGGAGACCGGCAGAT-	
**8**	CAGCACCTGTTGTTTGCATC	Confirmation of *THI21* deletion
	ACGCCAAAATCAGAACGAAG	
**9**	AATAGCTGCGCCGATGGTTTCTACA	Hygromycin amplification
	AACATCGCCTCGCTCCAGTCAATG	

Supporting Figures

Constrain-based stoichiometric model

The metabolic network of vitamin B1 in rice was constructed in a stepwise manner, by integrating genomic data gathered from MSU Rice Genome Annotation Project and National Center for Biotechnology Information (NCBI), and biochemical data through manual curation of an extensive survey of scientific literature and public databases such as KEGG, MetaCyc, Uniprot and BRENDA. Mass balance of the system was expressed as $\frac{d[c]}{dt} = S.v(t)$, where S corresponds to stoichiometric matrix and v(t) to the reaction rates. It was assumed that the system reached a steady state, therefore the metabolite balancing equation is reduced to $S.v(t) = 0$. COBRApy was used to carry out flux balance analyses, which uses the principles of linear programming to solve the system of mass balance equations by defining an objective function and searching the allowable solution space for an optimal flux distribution that maximizes or minimizes the objective. The constrains of the model were specified by applying lower and upper limit for the individual reactions $vmin \leq v \leq vmax$, where $vmin = 0$ for irreversible reactions. The stoichiometric model of *Oryza sativa* vitamin B1 pathway contains 16 reactions including external transport and inter-compartmental metabolite exchanges and 13. Aiming to analyze flux distributions in the metabolic pathways, we applied Flux Balance Analysis (FBA) with COBRApy.

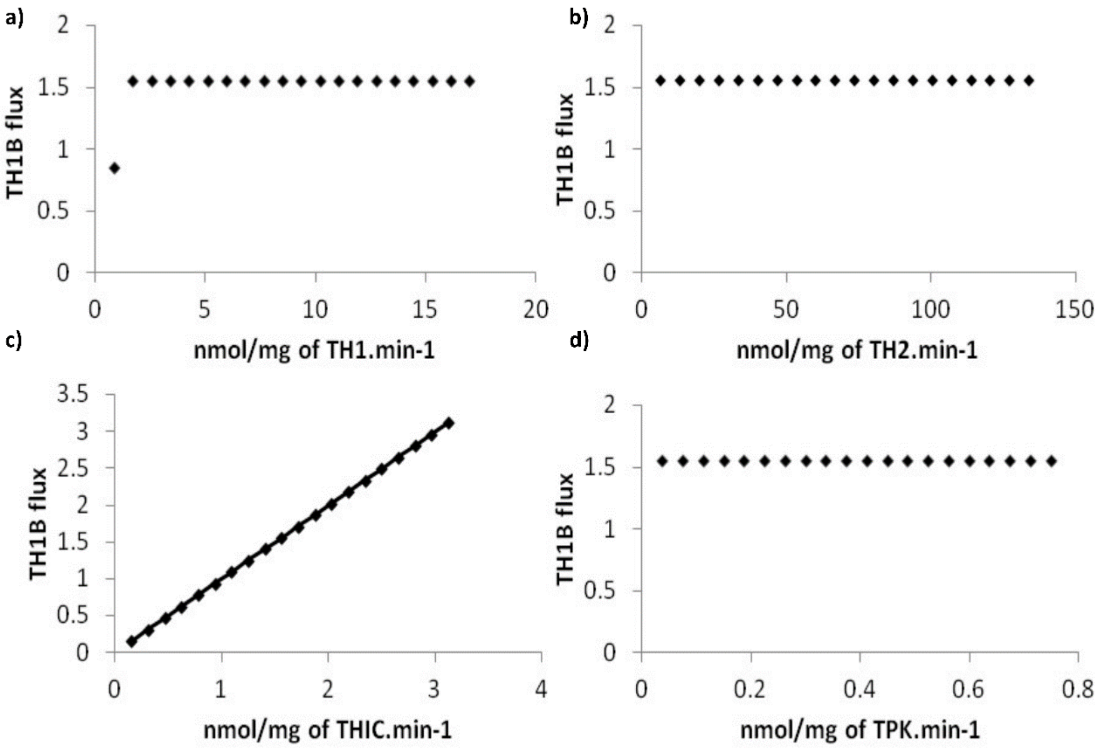

Figure S1. Solution to the FBA optimization problem maximization of TH1B flux. The objective function of the model was considered the maximization of thiamin monophosphate synthase (TH1B) flux. The correlation of each enzyme activity with the objective flux was carried out by setting the flux of each reaction as values from 0% to 200% of enzyme activity, with increments of 10%. Flux balance analysis (FBA) was run to simulate the optimal flux in TH1B. Reactions that had linear correlation with fluctuations in the objective flux were considered rate-limiting**. a)** Correlation between TH1 activity and TH1B flux. No correlation was observed. **b)** Correlation between TH2 activity and TH1B flux. No correlation was observed. **c)** Correlation between THIC activity and TH1B flux. THIC activity directly correlates with TH1B flux, pointing it as the limiting step of the pathway**. d)** Correlation between TTPK activity and TH1B flux. No correlation was observed.

References

1. Rapala-Kozik, M., Olczak, M., Ostrowska, K., Starosta, A. & Kozik, A. Molecular characterization of the *thi3* gene involved in thiamine biosynthesis in *Zea mays*: cDNA sequence and enzymatic and structural properties of the recombinant bifunctional protein with 4-amino-5-hydroxymethyl-2-methylpyrimidine (phosphate) kinase and thiamine monophosphate synthase activities. *Biochem J* **408,** 149–159 (2007).
2. Palmer, L. D. & Downs, D. M. The thiamine biosynthetic enzyme *ThiC* catalyzes multiple turnovers and is inhibited by S-adenosylmethionine (AdoMet) metabolites. *J. Biol. Chem.* **288,** 30693–30699 (2013).
3. Rapala-Kozik, M., Golda, A. & Kujda, M. Enzymes that control the thiamine diphosphate pool in plant tissues. Properties of thiamine pyrophosphokinase and thiamine-(di)phosphate phosphatase purified from *Zea mays* seedlings. *Plant Physiol Biochem* **47,** 237–242 (2009).
4. Yazdani, M., Zallot, R., Tunc-Ozdemir, M., de Crécy-Lagard, V., Shintani, D. K. & Hanson, A. D. Identification of the thiamin salvage enzyme thiazole kinase in Arabidopsis and maize. *Phytochem.* **94,** 68–73 (2013).

5. Martinis, J., Gas-Pascual, E., Szydlowski, N., Crèvecoeur, M., Gisler, A., Bürkle, L. & Fitzpatrick, T. B. Long-distance transport of thiamine (Vitamin B1) is concomitant with that of polyamines. *Plant Physiol.* **171,** 542–553 (2016).
6. Palmieri, L., Arrigoni, R., Blanco, E., Carrari, F., Zanor, M. I., Studart-Guimaraes, C., Fernie, A. R. & Palmieri, F. Molecular Identification of an Arabidopsis S-adenosylmethionine transporter. Analysis of Organ Distribution, Bacterial Expression, Reconstitution into Liposomes, and Functional Characterization. *Plant Physiol* **142,** 855–865 (2006).
